# Osteoporosis: are healthcare professionals missing an opportunity

**DOI:** 10.1186/2193-1801-2-463

**Published:** 2013-09-14

**Authors:** Yusra Habib Khan, Tauqeer Hussain Mallhi, Azmi Sarriff, Amer Hayat Khan

**Affiliations:** School of Pharmaceutical Sciences, University Sains Malaysia (USM), 11800 Pulau Penang, Malaysia; Government College (GC) University, 38000 Faisalabad, Pakistan

## Abstract

**Objectives:**

The objective of the current study is to analyze different approaches of pharmacists and general practitioners towards availability and use of osteoporosis prescreening tools and to find out reasons that explain non utilization of such tools in clinical practice.

**Settings:**

Among General practitioners and Community pharmacists in Pulau Penang, Malaysia.

**Methodology:**

An explorative cross sectional study was carried out using convenience sampling approach. A pre-validated self- administered questionnaire was used to carry out the study. A total of 170 healthcare professionals participated in study.

**Main outcome:**

Evaluation of awareness, use and opinions of healthcare professionals regarding osteoporosis prescreening tools.

**Results:**

Response rate of study was 56%. The mean age of the participants was 39.00 + 7.89 years. Less than one third of participants were familiar with term prescreening tools or Clinical decision rules. The only osteoporosis prescreening tool that was recognized and used by majority of participants was FRAX. Participants agreed that low level of awareness regarding availability of prescreening tools poses hindrance in utilization of such tools in clinical practice. Majority of participants showed willingness to gain information and use such tools in future.

**Conclusions:**

The results of our study demonstrate an urgent need of implementation of osteoporosis prescreening tools educational and awareness programs among healthcare professionals.

## Introduction

Osteoporosis is a metabolic bone disease that has potential to reach epidemic proportion. The increase prevalence of disease is attributed to low awareness of disease among general population and failure on part of healthcare professionals as far as diagnosis and management of disease is concerned (Freedman & Kaplan 2000; Chenot & Scheidt-Nave 2007). In order to increase awareness of osteoporosis in both general and diseased population, various studies have been conducted in different geographical settings (Yusra & Azmi 2012; Pauline & Chua 2013). Unfortunately, literature search shows paucity of data in terms of healthcare professionals’ role in both diagnosis and management of disease. This is evident from the fact that treatment rate for osteoporosis differs significantly from other medical conditions such as myocardial infarction for which 75% of patients receive appropriate treatment while only 10% of osteoporotic patients would receive adequate treatment (Giangregorio & Papaioannou 2006; Austin & Tu 2008). The reason for poor treatment rate of osteoporosis is attributed to the fact that unless a bone scan is done, it is impossible to diagnose osteoporosis. As DXA (Dual X-ray absorptiometry) is the gold standard for diagnosis of osteoporosis, extremely high cost and limited availability of DXA machines pose hindrance in timely diagnosis of disease and hence provision of appropriate treatment to patients suffering from osteoporosis (Gibson & Bogoch 2004). In order to cope up with current scenario, in the middle of 1990’s, various osteoporosis Clinical decision rules (CDRs) or pre-screening tools were developed. The purpose of such tools is to categorize individuals according to their risk status, to select most suitable candidates for bone scan and initiation of therapy according to level of risk e.g. dietary and lifestyle modifications in low to medium risk individuals while pharmacological therapy for one with high risk (Schwartz & Steinberg 2006; Elliott & Meek 2002). Ultimately use of osteoporosis prescreening tools would result in better utilization of bone densitometry, reduce economic burden associated with osteoporosis as well as improved patient quality of life (Tellier & Maeseneer 2001).

## Aim of study

Current study is the first study conducted in Malaysia with following aims:

- To analyze different approaches of pharmacists and general practitioners towards availability and use of osteoporosis prescreening tools
- To find out reasons that explain non utilization of such tools in clinical practice

## Methodology

As literature search did not show any study that explained opinions of health care professional regarding availability, awareness and use of osteoporosis pre-screening tools or explains reasons for non-implementation of such tools in clinical settings, therefore, a new questionnaire was developed to meet objectives of current study.

The questionnaire so developed was composed of two main parts. First part of questionnaire consisted of questions related to demographics i.e. age, race, gender, nationality, number of years in practice and specialty. The second part was composed of 11 questions regarding awareness, availability, utility, and possible reasons for non-implementation of existing pre-screening tools. All questions were close ended questions with options yes, no, don’t know. An excellent stability of questionnaire was obtained as Pearson’s *r* product moment correlation was 0.869.

Community pharmacies and General practitioners based in Penang and registered with Ministry of health, Malaysia were considered respondents for current study. Dillmans total design survey method was followed (Hoddinott & Bass 1986; Thorpe & Ryan 2009). Questionnaires along with consent form and a prepaid reply envelop were sent to participants via mailing. After three weeks of first mail, a reminder letter was sent to those who did not respond. Lastly a reminder letter was again sent in 7^th^ week for those participants who still did not respond back. A total of 300 questionnaires were sent i.e. 150 to each group. After 7^th^ week, total 170 questionnaires were received back making overall response rate of study as 56%. Statistical Package for Social Sciences (SPSS version 16) was used to analyze data.

## Results

The demographic profile of participants is shown in Table 1. Demographically majority of the study participants were male and belonged to Chinese ethnicity. First four questions were designed to assess awareness of CDRs while question 5, 6 and 7 were related to utility of CDRs. Opinions of health care professional regarding non-implementation of existing CDRs were assessed via question 8 while practices of healthcare professional, regarding implementation of CDRs in future, were asked in question 9 and 10. A list of different sources of information was given in last question and participants were requested to choose one source which they would like to use in order to gain information regarding CDRs. The frequency of responses for different questions in questionnaire is shown in Table 2. As clearly shown in Table 2, (80.5%) of participants were not aware about CDRs but surprisingly more participants were familiar with term prescreening tools. Participants were then asked to identify main purpose of prescreening tools. Only 30.7% were able to identify that prescreening tools are meant to identify or select risk group population. Out of list of 8 prescreening tools, the only prescreening tool that was identified by majority of participants was FRAX (Fracture risk assessment tool developed by WHO) while participants showed general lack of awareness towards other prescreening tools. Though only 10.7% of participants had utilized such tools in clinical practice yet majority of the participants were willing to gain information about such tools and use prescreening tools further in clinical settings. Majority of the participants (35.8%) agreed that the only barrier that poses hindrance in implementation of such tools is low level of awareness regarding availability and use of osteoporosis prescreening tools. Participants were lastly asked to select their preferred way by which they would like to gain information regarding osteoporosis prescreening tools and majority of them have chosen leaflets or brochures as their preferred way of gaining information. According to participants this mode is more convenient, flexible and less time consuming as compared to seminars and continuous education (CE) programs.

**Table 1:** **Demographic profile of healthcare professional (n = 170)**

Variable	N (%)	Mean ± SD
**Age**		39.00 ± 7.89
**Years in practice**		12.37 ± 8.24
**Gender**		
Male	63%	
Female	37%	
**Ethnicity**		
Malay	28%	
Chinese	48%	
Indian	24%	
**Nationality**		
Malaysian	100%	
Non-Malaysian	0%	
**Specialty**		
Pharmacists	53%	
General practitioners (GP’s)	47%

**Table 2:** **Frequency of responses to different parts of questionnaire**

Item no.	Yes		No	Don’t know
**1.** Have you ever heard about term Clinical Decision rules (CDRs)	19%		47%	34%
**2.** Have you ever heard about pre-screening tools which is an alternative term for CDRs	31%		29%	40%
**4.** Are you familiar/aware with following osteoporosis prescreening tools?				
**4a.** SCORE (simple calculated osteoporosis risk estimation)	7%		46%	47%
**4b.** OSIRIS (osteoporosis index of risk)	11%		37%	52%
**4c.** SOFSURF (study of osteoporosis fractures-study utilizing risk factors)	6%		37%	57%
**4d.** OSAT (osteoporosis self- assessment tool)	29%		29%	42%
**4e.** ORAI (osteoporosis risk assessment instrument)	0%		39%	61%
**4f.** WO-E (weight-only-EPIDOS)	1%		44%	55%
**4g.** MOST (Malaysian osteoporosis tool)	16%		31%	53%
**4h.** FRAX (Fracture assessment tool)	36%		23%	41%
**5.** If aware, have you ever utilized any osteoporosis prescreening tool? If yes, please mention name of tool:	11%		88%	1%
**6.** If utilized, do you think that use of such tools in clinical practice is practicable?	9%		2%	89%
**7.** Would you like to implement and use osteoporosis pre-screening tools in future?	47%		22%	31%
**9.** Would you like to aware general population about osteoporosis pre-screening tools?	53%		17%	30%
**10.** Would you suggest and motivate general population to undergo screening by such tools?	56%		16%	28%
**3.** According to you what is the main purpose of prescreening tools	**a**	**b**	**c**	**d**
**a.** To select high risk individuals	37%	12%	34%	17%
**b.** To educate general population about a disease				
**c.** To develop a care plan for patients				
**d.** None of the above				
**8.** According to you, what is the most common barrier in implementation of such tools?	**a**	**b**	**c**	**d**
**a.** Low level of awareness regarding availability of osteoporosis prescreening tools	39%	18%	28%	15%
**b.** Lack of knowledge regarding utilization of such tools				
**c.** Issues on reliability of results of such tools				
**d.** None of the above				
**11.** Which of the following ways would you prefer to gain knowledge about such tools?	**a**	**b**	**c**	**d**
**a.** Seminars	17%	16%	49%	18%
**b.** CE (continuous education) programs				
**c.** Leaflets/Brochures				
**d.** Internet				

## Discussion

A total of 170 healthcare professionals participated in current study making current response rate of study as 56%. The reason for low response rate might be attributed to mailing nature of survey rather than personnel interaction.

Majority (63%) were male while females represented 37% of study population. In order to assess awareness of osteoporosis CDRs, participants were first asked whether they have heard about CDRs or CPRs (Clinical prediction rules). They were then further asked whether they are familiar with the term pre-screening tools. Surprisingly, more number of participants showed awareness about prescreening tools. This shows the fact that although prescreening tools is an alternative term used for CDRs or CPRs, yet healthcare professional are more familiar with the term prescreening tools rather than CDRs or CPRs. According to Awareness to Adherence model which was originally proposed by Pathman et al in 1996, awareness is the first and foremost step that would compel an individual to practically implement knowledge into clinical practice (Pathman & Konrad 1996; Heneghan & Perera 2007). As seen in our study only less than one third of healthcare professionals were aware of availability of osteoporosis CDR’s or pre-screening tools, this explains one of the major reason for non-utilization of pre-screening tools in clinical practice. Even majority of participants were not able to identify main purpose of pre-screening tools. This again shows lack of awareness and main hindrance in practical implementation of such tools. FRAX was the only osteoporosis prescreening tool that was identified by majority of participants and surprisingly the only tool that was being used in clinical practice. In past, various studies have been conducted among healthcare professional regarding adoption of clinical guidelines. Results of such studies have shown that one of the main reasons that healthcare professional would not adopt guidelines or wished to know about guidelines would be lack of confidence in developers of guidelines or recommendations (Hayward & Guyatt 1997; Carlsen & Norheim 2008). The possible reason for awareness of FRAX as pre-screening tool of osteoporosis might be due to increase trust of healthcare professional in developers of FRAX, as FRAX was developed by WHO. As WHO is a worldwide trusted organization and also FRAX is the only pre-screening tool that has been included in Clinical guidance in management of osteoporosis published by Malaysian osteoporosis foundation (2012), this might explains its increase awareness, as compared to other prescreening tools, among healthcare professional.

Mann–Whitney U test was used to observe differences between scores of pharmacists and GPs. A p-value of less than 0.05 was considered statistically significant. For this purpose, each correct answer, positive attitude towards CDR use or knowledge regarding existing Clinical decision rules was given one mark. On the other hand, incorrect answers, negative answer (no) or undecided answers (don’t know) were given zero mark. Question number 8 was exempted from this scoring system as there is no well documented reason for non-utility of Clinical decision rules, so participants can have their own opinions. There was a statistically significant difference between mean rank score of both groups with pharmacists scoring a mean rank score of 137.55 while GP’s scoring a mean rank score of 78.05. A comparison of responses of both groups to towards awareness and use of CDRs’ is shown in Table 3. It clearly shows that as compare to GPs’, pharmacists are not only well aware about prescreening tools but they are also more willing to implement such tools in clinical practice.

**Table 3:** **Comparison of frequency of responses of pharmacist and physicians**

Item no	Pharmacist group				GPs’ group			
	Yes	No/Don’t know			Yes	No/Don’t know		
**1**	23%	77%			13%	87%		
**2**	43%	57%			17%	83%		
**4a**	10%	90%			4%	96%		
**4b**	15%	85%			5%	95%		
**4c**	6%	94%			5%	95%		
**4d**	34%	66%			22%	78%		
**4e**	0%	100%			0%	100%		
**4f**	2%	98%			0%	100%		
**4g**	20%	80%			10%	90%		
**4h**	46%	54%			24%	76%		
**5**	16%	84%			5%	95%		
**6**	14%	86%			2%	98%		
**7**	49%	51%			44%	56%		
**9**	61%	39%			43%	57%		
**10**	65%	35%			45%	55%		
**3**	**3a**	**3b**	**3c**	**3d**	**3a**	**3b**	**3c**	**3d**
	48%	8%	42%	1%	24%	16%	24%	35%
**8**	**8a**	**8b**	**8c**	**8d**	**8a**	**8b**	**8c**	**8d**
	47%	21%	13%	18%	29%	15%	45%	10%
**11**	**11a**	**11b**	**11c**	**11d**	**11a**	**11b**	**11c**	**11d**
	12%	23%	38%	26%	22%	7%	61%	9%

Overall healthcare professional showed poor knowledge regarding availability and use of osteoporosis pre-screening tools. But good part is that they were willing to develop positive perspective towards pre-screening tools as they were interested to use them in clinical practice. According to trans-theoretical model (TTM), various stages are involved in adoption of a new behavior, strategy or recommendation (Velicer & Prochaska 1998). First stage is known as pre-contemplation phase. In this stage an individual does not have sufficient awareness regarding concerned issue. As seen in our study that health care professional were unaware about availability of osteoporosis pre-screening tools even they were unable to recognize what are pre-screening tools and why they are used. Second phase is the contemplation phase, at this stage individual gets introduced to concerned issue. In current study, the conduction of such study among healthcare professional shifts healthcare professional from pre-contemplation phase to contemplation phase. Third phase in trans-theoretical model is known as preparation phase. Individual will show willingness to adopt new behavior towards concerned issue in this phase. In our study, majority of healthcare professional showed willingness to use such tools in future. Next stage is the most important stage in TTM i.e. action stage followed by maintenance phase. The need of the hour is to educate healthcare professionals regarding availability and use of such tools so that they can enter action stage of TTM and utilize prescreening tools in clinical settings.

## Conclusions

Osteoporosis prescreening tools are widely available pre-validated tools that can be utilized in clinical practice by healthcare professionals in order to select high risk individuals and save cost associated with conventional bone scanning. As healthcare professionals are currently unaware about availability and use of such tools therefore, future studies should focus on creating awareness and educating healthcare professionals about these tools rather than development of new osteoporosis pre-screening tools.

## References

[CR1] Austin PC, Tu JV (2008). Use of evidence-based therapies after discharge among elderly patients with acute myocardial infarction. Canadian Medical Association Journal.

[CR2] Carlsen B, Norheim OF (2008). What lies beneath it all?" – an interview study of GPs' attitudes to the use of guideline. BMC Health Services Research.

[CR3] Chenot R, Scheidt-Nave C (2007). "German primary care doctor’s awareness of osteoporosis and knowledge of national guidelines". Experimental and Clinical Endocrinology & Diabetes.

[CR4] Elliott M, Meek P (2002). Pharmacy-based bone mass measurement to assess osteoporosis risk. The Annals of Pharmacothery.

[CR5] Freedman KB, Kaplan FS (2000). "Treatment of osteoporosis: Are physicians missing an opportuinity?". The Journal of Bone & Joint Surgery.

[CR6] Giangregorio L, Papaioannou A (2006). Fragility fractures and the osteoporosis care gap: an international phenomenon. Seminars in Arthritis and Rheumatism.

[CR7] Gibson VE, Bogoch E (2004). Practice patterns in the diagnosis and treatment of osteoporosis after a fragility fracture: a systemic review. Osteoporosis International.

[CR8] Hayward R, Guyatt G (1997). Canadian physicians' attitudes about and preferences regarding clinical practice guidelines. Canadian Medical Association Journal.

[CR9] Heneghan C, Perera R (2007). Hypertension guideline recommendations in general practice: awareness, agreement, adoption, and adherence. The British Journal of General Practice.

[CR10] Hoddinott SN, Bass MJ (1986). The Dillman Total Design Survey Method. Canadian Family Physician.

[CR11] Pathman D, Konrad T (1996). The awareness-to-adherence model of the steps to clinical guideline compliance. The case of pediatric vaccine recommendations. Medical Care.

[CR12] Pauline SML, Chua SS (2013). Impact of pharmaceutical care on knowledge, quality of life and satisfaction of postmenopausal women with osteoporosis. International Journal of Clinical Pharmacy.

[CR13] Schwartz EN, Steinberg DM (2006). Prescreening tools to determine who needs DXA. Current Osteoporosis Report.

[CR14] Tellier V, Maeseneer JD (2001). Intensive and prolonged health promotion strategy may increase self-reported osteoporosis prevalence among postmenopausal women. Osteoporosos International.

[CR15] Thorpe C, Ryan B (2009). How to obtain excellent response rates when surveying physicians. Family Practice.

[CR16] Velicer WF, Prochaska JO (1998). Smoking cessation and stress management: Applications of the Transtheoretical Model of behavior change. Homeostatis.

[CR17] Yusra HK, Azmi S (2012). “A knowledge, attitude and practice survey of osteoporosis among community population”. J App Pharm.

